# A Liquid–Solid Interface-Based Triboelectric Tactile Sensor with Ultrahigh Sensitivity of 21.48 kPa^−1^

**DOI:** 10.1007/s40820-022-00831-7

**Published:** 2022-04-01

**Authors:** Jingya Liu, Zhen Wen, Hao Lei, Zhenqiu Gao, Xuhui Sun

**Affiliations:** Institute of Functional Nano and Soft Materials (FUNSOM), Jiangsu Key Laboratory for Carbon-Based Functional Materials and Devices, Soochow University, Suzhou, 215123 People’s Republic of China

**Keywords:** Ferrofluid, Tactile sensor, Triboelectric nanogenerator, Microstructure, Ultrahigh sensitivity

## Abstract

Traditional triboelectric tactile sensors based on solid–solid interface have illustrated promising application prospects through optimization approach. However, the poor sensitivity and reliability caused by hard contact-electrification still poses challenges for the practical applications. In this work, a liquid–solid interface ferrofluid-based triboelectric tactile sensor (FTTS) with ultrahigh sensitivity is proposed. Relying on the fluidity and magnetism of ferrofluid, the topography of microstructure can be flexibly adjusted by directly employing ferrofluid as triboelectric material and controlling the position of outward magnet. To date, an ultrahigh sensitivity of 21.48 kPa^−1^ for the triboelectric sensors can be achieved due to the high spike microstructure, low Young’s modulus of ferrofluid and efficient solid–liquid interface contact-electrification. The detection limit of FTTS of 1.25 Pa with a wide detection range to 390 kPa was also obtained. In addition, the oleophobic property between ferrofluid and poly-tetra-fluoro-ethylene triboelectric layer can greatly reduce the wear and tear, resulting in the great improvement of stability. Finally, a strategy for personalized password lock with high security level has been demonstrated, illustrating a great perspective for practical application in smart home, artificial intelligence, Internet of things, etc.

**Supplementary Information:**

The online version contains supplementary material available at 10.1007/s40820-022-00831-7.

## Highlights

- A self-powered liquid–solid interface ferrofluid-based triboelectric tactile sensor with immediately formed spike-shaped microstructure is proposed.
- Due to the high spike microstructure, low Young’s modulus of ferrofluid and efficient solid–liquid interface contact-electrification, an ultrahigh sensitivity of 21.48 kPa^−1^ for the triboelectric sensors can be achieved.
- A strategy for personalized password lock with high security level is demonstrated.

**Supplementary Information:**

The online version contains supplementary material available at 10.1007/s40820-022-00831-7.

## Graphical abstract

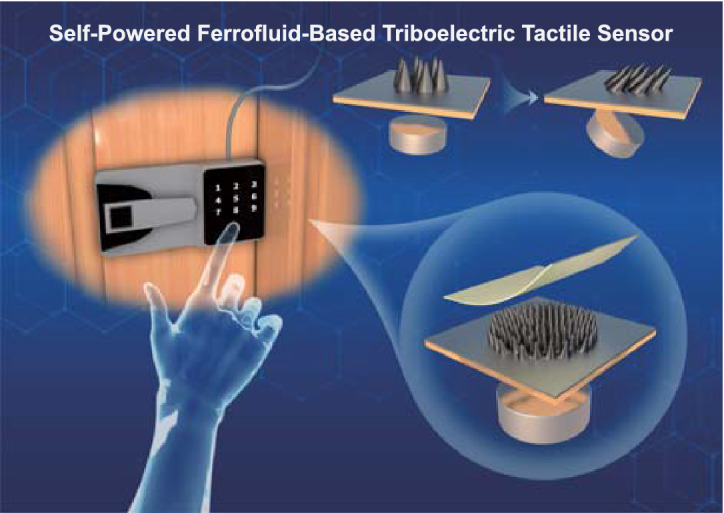

## Introduction

With the rapid development of Internet of Things, sensing technology has gradually become an indispensable part of people's daily life served for various purposes [1–5]. Triboelectric nanogenerator (TENG) based on the coupling effects of contact electrification and electrostatic induction [6–9] has laid a great foundation for triboelectric sensor and has been applied in many aspects [10–12]. According to Hook’s law, the voltage output of TENG is linear to the applied force, thus showing high linearity of the detection [13]. In addition, triboelectric sensor also has the advantages of detecting both static and dynamic pressure, simple fabrication, wide material choice and broad application potential. However, the sensitivity of triboelectric sensor is not high enough at present [14, 15].

Triboelectric sensor is one of the most common and important types of tactile sensors [16]. It has been known that the geometry of the active layer has a significant influence on a sensor’s performance [17]. Therefore, a plenty of work in microstructure engineering has been done recently to improve the sensitivity of the sensor [18–23]. Nevertheless, the fabrication methods of microstructure are mostly through photolithography, 3D printing technology and molding, etc. These methods are not only complicated but also costly. Furthermore, when each device is made, the generated microstructure is fixed and the sensitivity will no longer change. To change the sensitivity, people need to reconstruct the microstructure from scratch, it takes a lot time and effort. In addition, hard contact will cause wear and tear in the traditional solid/solid triboelectric models, thus lead to the decrease in stability.

In this work, a simple and low-cost strategy to create a liquid–solid interface-based triboelectric tactile sensor with ultrahigh sensitivity based on ferrofluid is proposed by employing the special characteristics of ferrofluid [24–28]. The ferrofluid itself is directly used as a liquid triboelectrification layer. When a magnet is placed, the ferrofluid immediately forms a great deal of spike structures under the influence of an external magnetic field. As the distance or angle of the magnet position changes, the morphology of spikes will also change. Therefore, the sensor’s sensitivity can be achieved dynamically and in real-time simply by changing the position of the magnet to control the topography of microstructure. A maximum sensitivity of 21.48 kPa^−1^ has been realized when the distance between ferrofluid and magnet is 12 mm, which can be attributed to high spike-shaped microstructure and low Young's modulus of ferrofluid. By flexibly applying the combination of this ferrofluid-based triboelectric tactile sensor (FTTS), it can be used as personalized password lock with variable force requirement to enhance security level.

## Experimental Section

### Materials

Organic-based ferrofluid (Ink king, Japan) was purchased directly from Franchiser. NdFeB magnet (*D* = 10 mm) was bought from YPE store. All reagents were used as received without further purification.

### Fabrication of the FTTS

The acrylic plate was produced by a laser cutting machine as a supporting layer. Aluminum foil is attached as a single bottom electrode. The ferrofluid is injected onto the aluminum foil as the positive friction layer, and poly-tetra-fluoro-ethylene (PTFE) film is used as negative layer. A permanent magnet is used to provide the external magnetic field.

### Characterization and Measurement

A Leica optical microscope was used to characterize the detailed structure of ferrofluid. The characterization of PTFE was obtained through a scanning electron microscope (ZEISS G500) and a contact angle meter. The electrical output performance of TENG including *V*_oc_, *I*_sc_, and *Q*_tr_ was tested by a programmable electrometer (Keithley 6514), and the real-time data acquisition was realized by a software platform constructed based on LabView. A vertical single-axis motor (HC14-10) was used to apply variable vertical force, which was measured by a pressure sensor (DS2-2000 N-XD). The video was shot by Canon Camera. A numerical gauss meter was served to measure magnetic field intensity.

## Results and Discussion

### Characteristics of Ferrofluid

**Fig. 1:**
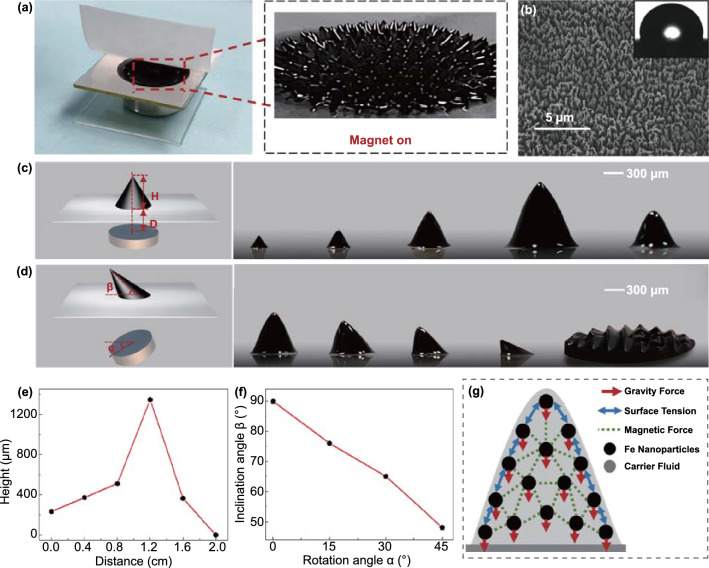
Dynamic behaviors of the ferrofluid. **a** Photograph of FTTS. Inset displays the topography of microstructure in response to magnetic field. **b** The SEM image of PTFE membrane used to contact with ferrofluid, scale bar 5 μm. Inset shows the photograph of its contact angle. Schematic illustration and real-time microscope images of the dynamic behaviors of the ferrofluid in response to changing **c** distance and **d** angle with the magnet, scale bar 300 μm. Quantitative relations between different magnet conditions and geometric parameters of ferrofluid spikes: **e** distance and height, **f** rotation angle and inclination angle. **g** Force analysis of ferrofluid under magnetic field

Figure 1 generally illustrates the dynamic behaviors of the ferrofluid. The photographs of FTTS and the microstructure formed by the ferrofluid in response to the magnetic field are shown in Fig. 1a. A lot of spikes are distributed along the magnetic induction line from the inset. PTFE film was chosen as another triboelectrification material to contact with the ferrofluid because of its high stability, oleophobicity and charge-generation capability. Besides, it has rich fluorine groups and low surface energy. Figure 1b displays the scanning electron microscopy (SEM) image of PTFE surface morphology and the contact angle (101.4°). Figure 1c, d exhibits the schematic illustrations and real-time microscope images of the dynamic behaviors of the ferrofluid in response to variable distance (D) and rotation angle (α) with the magnet, respectively. The corresponding height and inclination angle of single ferrofluid spike are H and β. Furthermore, the quantitative relations between different magnet conditions and geometric parameters of the ferrofluid spikes are demonstrated in Fig. 1e, f. At 0.4 cm intervals of D, it can tell that the height of a single spike reaches the peak at 1.2 cm and at this time the height of the spike exceeds 1 mm, then the height gradually decreases until it disappears completely at 2.0 cm. Nevertheless, at 15° intervals of α, the inclination angle of a single spike keeps decreasing from the beginning 90° as the rotation angle increases. And there is no one single spike but stacked ferrofluid when the rotation angle reaches 60°, this can be explained in Fig. 1g which indicates the force analysis of ferrofluid under magnetic field [29].

Ferrofluid is composed of nanoscale ferromagnetic particles suspended in the carrier fluid, and the carrier fluid is usually organic solution or water. The ferromagnetic particles are encapsulated by surfactants to prevent their condensation due to van der Waals force and magnetic force. Ferrofluid has no magnetic attraction in static state. However, once an external magnetic field is imposed, the ferrofluid shows magnetism that the magnetic nanoparticles are well suspended. In this case, the magnetic nanoparticles will retain the same topography and not disperse over time provided there is a magnetic field all the time. As shown in Fig. 1g, when the magnetic field is strong enough, magnetic force can overcome the surface tension and gravity force, leading to a phenomenon of normal-field instability [30], which refers smooth surface naturally forms folds. To evaluate local magnetic forces, many physical equivalences and analytical formulas derived from magnetostatics can well estimate the overall force of ferrofluid under an external magnetic field. The kelvin force formulations are commonly used in hydrodynamics. Many previous works used Kelvin's law (Eq. 1) to calculate the force density [31].

1 $$f = \mu_{0} \left( {M \cdot \nabla } \right)H$$

where *μ*_0_ is the vacuum permeability [H m^−1^], *H* is the magnetic field in the material [A m^−1^], *M* is the magnetization of the material [A m^−1^]. But it has gradually come to people’s sense that Eq. (1) is not accurate enough to represent the total force density. Further consideration of surface factor is needed to acquire the correct formula as in Eq. (2):

2 $$F = \int\limits_{V} {\mu_{0} \left( {M \cdot \nabla } \right)H{\text{d}}V} + \oint\limits_{S} {\frac{{\mu_{0} }}{2}\left( {M_{n} } \right) ^{2} {\text{d}}S}$$

where *Mn* is the normal magnetization vector at the surface of ferrofluid. By applying the Green–Ostrogradski theorem to explain the above formula, the expression of local force can be obtained as in Eq. (3):

3 $$f = \mu_{0} \left( {M \cdot \nabla } \right)H + \frac{{\mu_{0} }}{2}\nabla \cdot \left( {M^{2} } \right)$$

when the rotation angle is 60°, the magnetic force is too small to support ferrofluid to form distinct spike-like microstructure.

### Structure and Properties of FTTS

**Fig. 2:**
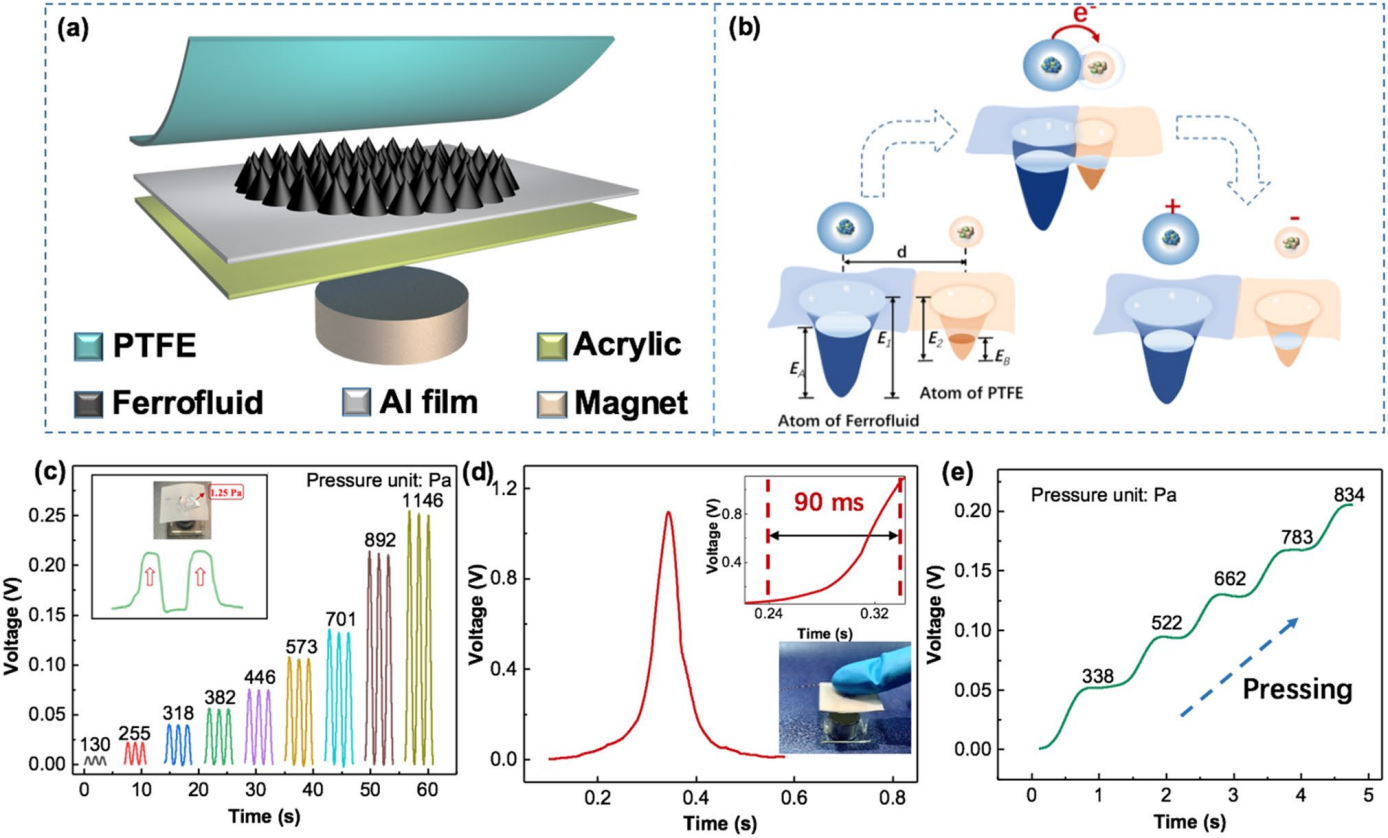
Basic structure and sensing properties of FTTS. **a** Schematic illustration of the FTTS. **b** Explanation of contact electrification phenomenon by electron cloud interaction. **c** Output voltage of the FTTS under varying static pressures imposed by copper probe. The inset shows low detection limit of 1.25 Pa. **d** Response time of the FTTS. The inset above is a partial enlargement of the curve. The inset below is demonstration of pressing. **e** Real-time output voltage changing under dynamic variable pressure

The schematic diagram of the structure of FTTS is shown in Fig. 2a. The TENG model in this pressure sensor is a simple single-electrode TENG which includes three parts: a PTFE film and the ferrofluid for contact electrification, an aluminum film for electrostatic induction electrode and a polymethyl methacrylate (PMMA) plate for supporting substrate. And the magnet is introduced to control the ferrofluid’s microstructure through changing the magnetic field strength. Figure 2b illustrates the contact electrification phenomenon between the ferrofluid and PTFE by establishing the electron cloud interaction model. Atoms are represented by a potential well. Electrons occupy specific atomic orbits and bind loosely together in the potential well to form electron clouds. In Fig. 2b, *d* is the distance between electron clouds, *E*_1/2_ is the potential energy required for electrons to escape from the material, and *E*_A/B_ is the energy level occupied by electrons in the material atom, which is obviously less than the former. Before the two materials contact, the electrons cannot transfer because of the potential wells’ local trapping effect. When the two materials are in contact, the electron clouds would overlap due to the screening of the two materials, then the original single potential well turns into an asymmetric double-well potential, it creates condition for the electron to transfer from the atom of one material to the atom of another material. After two materials separating, the transferred electrons will be mostly remained due to the existing energy barrier in material as long as the external condition, such as temperature, do not change. The complete working principle of the FTTS is demonstrated in Fig. S1, thanks to the existence of fluorine and oxygen atoms with great surface electron affinity in the chemical composition of PTFE, it tends to attract the electrons from the ferrofluid after contact, which makes PTFE film is with negative charge and ferrofluid is with positive charge (state I). As the pressure releasing, positive triboelectric charges in ferrofluid will drive the electrons to flow from the ground to the Al electrode (state II). When no pressure applied, an equilibrium is achieved on the electrode and no electrical signal is observed at this time (state III). As the pressure pressing, the induced electrons flow back to the ground to balance the potential change on the electrode (state IV). The electrical performance of this device is measured in contact separation mode driven by a linear motor is shown in Fig. S2. As the frequency increases from 0.5 Hz to 3 Hz, the short-circuit current (*I*_sc_) increases almost linearly, while the open circuit voltage (*V*_oc_) and short-circuit charge transfer quantum (*Q*_tr_) remain almost unchanged. Moreover, the device shows excellent stability and robustness after 10,000 test cycles of continuously loading and unloading because the solid–liquid interface greatly reduces the abrasive wear between the triboelectric layers (Fig. S3). These above electrical output results have laid a solid foundation for this device to be used as triboelectric tactile sensor.

The static pressure sensing performance of the FTTS is presented in Fig. 2c. The probe of the pressure test platform used to apply pressure on device is remade of copper because the original stainless steel one will affect the ferrofluid due to the existence of magnetism. It could be observed that the output voltage increases with increasing pressure from 130 Pa to 1146 Pa, and the detection limit of the FTTS for dynamic sensing is as low as 1.25 Pa. Also, FTTS possesses fast response time of ~ 90 ms with one gentle touch (Fig. 2d). In order to further investigate its sensing characteristics, the dynamic pressure sensing of the FTTS is shown in Fig. 2e. Under the same loading rate, the measured voltage output exhibits a clearly increasing trend with gradually elevated pressures, and the existence of the platform is because of the stop of the copper probe motion. To sum up, the device can realize active sensing in both static and dynamic stimuli condition.

### Pressure Sensing Performance of FTTS

Because of the close relationship between open circuit voltage (*V*_oc_) and the sensitivity of FTTS, it is of huge significance to study the parameters affecting *V*_oc_ and then infer the factors that affect the sensing performance. Usually, the open circuit voltage can be defined as:

4 $$V_{{{\text{OC}}}} = \frac{\sigma d}{{\varepsilon_{0} }}$$

where *ε*_0_ is the permittivity in vacuum and *σ* is the triboelectric density, *d* is the distance between the top and bottom components of TENG and equals to the height of the supporting structure between the two components, respectively. In this case, *d* can be regarded as the height of the spike microstructure when the ferrofluid is employed under external magnetic field. On the other hand, sensitivity which is the most important parameter of pressure sensor is generally expressed as follows:

5 $${\text{S}} = \frac{{\partial V_{{{\text{OC}}}} }}{\partial P}$$

where ∂*V*_oc_ is the open circuit voltage’s responding to the change of pressure (∂*P*). According to Hook's law, the material elasticity of FTTS is counted as the spring-entangled structure, then we can obtain the sensitivity as:

6 $$S = \frac{\sigma }{{\varepsilon_{0} }} \cdot \frac{d}{Y}$$

where *Y* is the Young’s modulus of the intermediate object. It is obvious that the sensitivity is positively correlated with *d/Y* and high *d* and low *Y* can contribute to the sensitivity improvement [32]. Nevertheless, previous report pointed out that there is a limitation of enhancing *d*, *d* cannot be enhanced infinitely. When *d* reaches to a certain value, more enhancement could not make much a difference, which means the transferred charges cannot be raised effectively anymore, instead will damage the conformity of the device. Besides, lower *Y* is beneficial to improving sensitivity too, yet may lead to the impair of sensing pressure range.

**Fig. 3:**
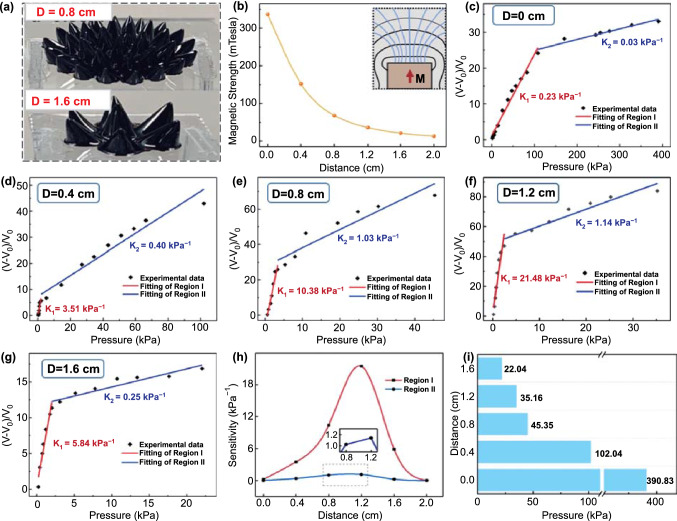
Pressure sensing performance under different distances (*D*) with magnet. **a** Photograph of diverse formations of ferrofluid at different distances. **b** Characterization of the magnetic field strength generated under different distances. The inset shows the magnetic induction line in the direction of distance change. **c-g** The summarized relationship and linear fitting between the relative variations of voltage and the pressure applied on the device under different distances with magnet, from 0 to 16 mm at 4 mm intervals. The summarized variation of **h** sensitivity and **i** detection range of pressure sensor under different distances with magnet

Figure 3a displayed the photographs of the ferrofluid when the distance with permanent magnet is 0.8 cm and 1.6 cm, respectively. Characterization of the magnetic field strength under different distances from 0 to 2 cm is depicted in Fig. 3b; the inset is diagram of the magnetic induction line in the vertical direction. We can conclude that the magnetic field strength decreases with the increase in distance in the vertical direction and nearly disappears at 2 cm. Figure S4 complementally presents the topography of the ferrofluid patterns at all six distances, from 0 to 2 cm at intervals of 4 mm. It should be noted that with the weakening of magnetic field strength, the height of formed spike microstructure first increases until reaches the maximum at the distance of 12 mm, then decreases until it disappears. But in the whole process, the ferrofluid keeps becoming more and more sparse in density. The overall height trend is consistent with Fig. 1c. The relationship between relative voltage change (*V*-*V*_0_)/*V*_0_ and the magnitude of pressure at different distances (*D* = 0, 4, 8, 12, 16 mm) is revealed in Fig. 3c–g, and the fitting slope of the plot represents the sensitivity of the sensor. On the basis of the difference of sensitivity, these plots can be separated into two regions. The possible explanation for the distinction of two regions is proposed according to the theoretical derivation: in the low-pressure region, the increase in pressure will cause the obvious variation of *d* between the two friction layers (*d* is the height of the ferrofluid spike), so the corresponding *V*_oc_ also changes greatly; when the pressure increases to a certain extent, increasing the pressure will not change *d* apparently, and the effective contact area is only slightly increased, so the *V*_oc_ changes slowly and the sensitivity will drop a lot. The summarized variation curve of sensitivity in both regions is presented in Fig. 3h. It should be emphasized that the sensitivity is maximum for both regions at *D* = 12 mm and reach up to 21.48 kPa^−1^ and 1.14 kPa^−1^ in the pressure range of 0–2.5 kPa and 2.5–35 kPa, respectively. The experimental results are in good agreement with the theoretical derivation, because when *D* = 12 mm, the shape of the spike microstructure is the highest (more than 1 mm), and naturally the sensitivity of the device is also the highest. At the same time, the fluidity of ferrofluid endows it a relatively small Young's modulus, which also contributes to the ultrahigh sensitivity (Fig. 3f). Figure 3i summarizes the variation of detection range of FTTS device (*D* = 0, 4, 8, 12, 16 mm), we can see that as the distance increases, the magnetic field intensity diminishes and the total pressure detection range decreases from 390 kPa to nearly 20 kPa.

**Fig. 4:**
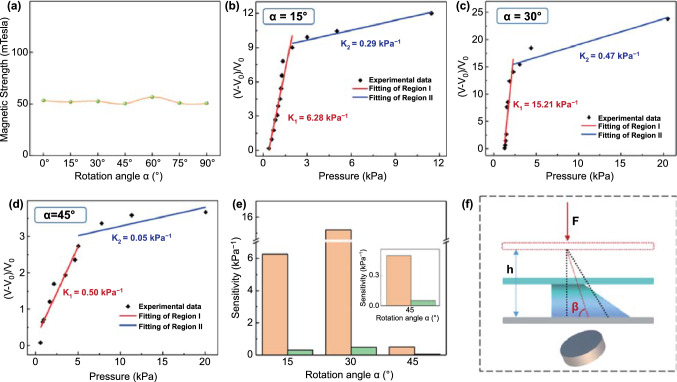
Pressure sensing performance under different angles (α) with magnet. **a** Characterization of the magnetic field strength generated under different angles. **b-d** The summarized relationship and linear fitting between the relative variations of voltage and the pressure applied on the device under different angles with magnet, from 15° to 45°at 15° intervals. **e** The summarized variation of sensitivity of pressure sensor under different distances with magnet. **f** Force analysis diagram when tilted ferrofluid spike is pressed

In addition to the distance factor with the permanent magnet, the angle factor was also studied. Characterization of the magnetic field strength under different angles from 0° to 90° is depicted in Fig. 4a, which indicates the magnetic strength at the same position (*D* = 4 mm) is almost constant with the change of rotation angle. However, in Fig. 1d, the spike microstructure becomes inclined and also weakened with the increase in the rotation angle, and even not able to form an independent spike at the rotation angle of 60°. This is because only when the ferrofluid is in a strong enough vertical magnetic field can its surface form a microstructure; however, in the circumstance of the permanent magnet rotating, the magnetic field in the vertical direction becomes weaker and weaker as the rotation angle increasing until the spike disappears. The relationship between relative voltage change (*V*−*V*_0_)/*V*_0_ and magnitude of pressure at three diverse rotation angles (α = 15°, 30°, 45°) is depicted in Fig. 4b–d. Compared with the initial position (Fig. 3d), the sensitivities of both regions improve as the rotation angle increases to 30° where the sensitivity reaches up to 15.21 kPa^−1^ in region I and 0.47 kPa^−1^ in region II, yet drop a lot at α = 45° where the sensitivities are merely 0.5 kPa^−1^ in region I and 0.05 kPa^−1^ in region II, this variation trend is revealed in Fig. 4e. We think that the promotion of FTTS’s sensitivity in the early stage is due to the increase in contact area when the inclined spike microstructure is compressed. To illustrate this phenomenon, Fig. 4f presents force analysis diagram when tilted ferrofluid spike is pressed. The dotted line represents the inclined spike before compression, the colored part shows the inclined spike under compression. It is obvious that the inclined spike-structure cause bigger contact area, resulting in higher charge density and sensitivity. In addition, when the rotation angle reaches 45°, the influence of gravity exceeds the influence of magnetic field in the vertical direction, the ferrofluid microstructure is too small and weak; hence, the sensitivity is greatly reduced.

### Application as a Password Lock

**Fig. 5:**
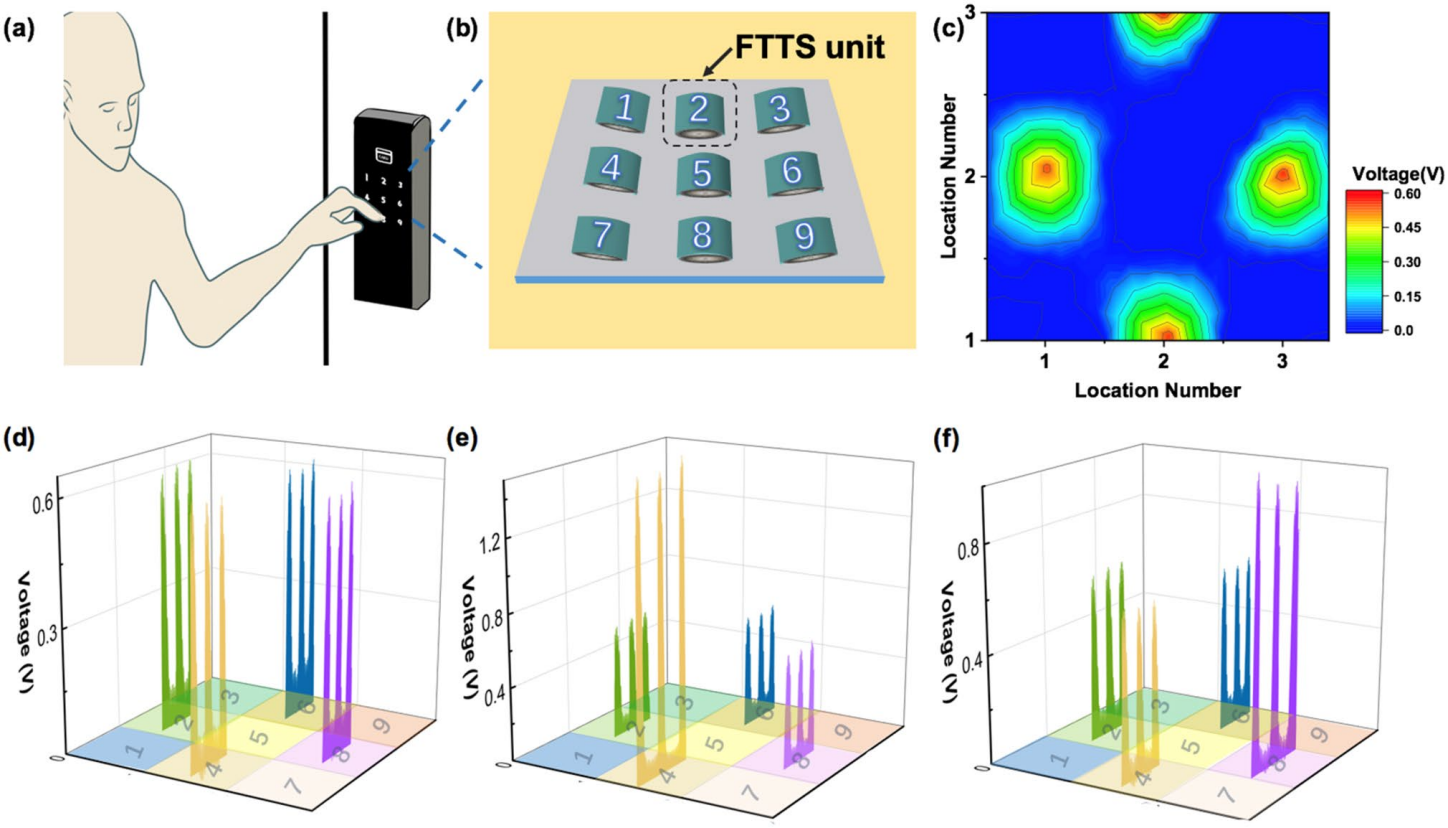
Application as novel password lock with variable force requirement. **a** The schematic illustration of people unlocking password lock. **b** FTTS worked as nine-digit password lock. **c** The contour map of voltage with password “2468” under the same external pressing. The three-dimensional diagram of voltage with password “2468” under the same external pressing with **d** all the same D = 0 mm, **e** one different D = 4 mm at “4”, **f** one different D = 16 mm at “8”

With the development of economy and the improvement of people's living standard, password lock is more and more popular, due to the great convenience of not having to carry a key around. However, most of the current password lock can be opened by anyone knowing the password combination, which is dangerous if the password is maliciously obtained by others. On the basis of FTTS, a compound password lock is provided. By controlling the pressure when inputting the password combination, the double protection of the password lock is formed, which overcomes the shortcoming that the existing password lock is easy to leak the password and brings absolute safe and reliable protection to the user. Figure 5a shows one person unlocking the password lock by pressing number combination, and the array of FTTS units was fabricated as schematically illustrated in Fig. 5b. The photograph of FTTS array as a nine-digit password lock is exhibited in Fig. S5. Every unit is marked with the numbers 1 to 9, and their electrodes were connected to the probe for output measurement. A responsive voltage peak will be recorded as a local pressure applied on the relevant FTTS unit. The voltage output of all FTTS units in the matrix is at the background level before any pressing as displayed in Fig. S6. And Fig. 5c presents the two-dimensional contour plot of the peak value of the voltage responses that were measured when external pressures were applied in accordance with password combination “2468,” the highlighted color indicates the area pressed through different number. This plotting elaborates the spatial resolution of the FTTS matrix for distinguishably mapping the password of the applied pressure. Figure 5d demonstrates the three-dimensional diagram of voltage output with password “2468” under the same external pressing (*F* = 20 N), all the FTTS units are in the initial position (*D* = 0 mm) at this time. By taking the advantage of FTTS, we can adjust the distance of one specific individual unit in the corresponding password combination. The three-dimensional diagram of voltage with password “2468” under the same external pressing with one different *D* = 4 mm at “4” and one different *D* = 16 mm at “8” is depicted in Fig. 5e, f. Figure S7 demonstrates different voltage output while applying the same external force (*F* = 20 N) on same FTTS unit through changing the distance with magnet. As we all know, everyone has his/her own different habit of exerting and would not changing force at specific spot during pressing password generally. Through making use of the characteristics of FTTS, we can achieve personalized force requirements when inputting password: for instance, we can set a string of digital password combination such as “1397,” which demands pressing 9 harder to achieve ideal output to unlock. Therefore, by simply adjusting the distance, we can realize a variety of pressure requirements of one password combination, which greatly improves the security of the password lock. The demo of owner unlocking and imposters unlocking failed is demonstrated in Video S1. Better yet, it has been reported that the magnetic property of ferrofluid can also be used for sensing, then it is likely for FTTS to monitor the door’s opening times or other actions. In the future, it has huge potential in smart home and Internet of things.

## Conclusion

In summary, a liquid–solid interfacing triboelectric tactile sensor with ultrahigh sensitivity of 21.48 kPa^−1^ based on ferrofluid is presented. This ultrahigh sensitivity can be attributed to the high microstructure, low Young’s modulus of ferrofluid as triboelectric material and efficient solid–liquid interface contact-electrification. By employing the special characteristics of fluidity and magnetism of the ferrofluid, the topography of spike microstructure can be flexibly adjusted by controlling the position of outward magnet. We studied two factors including magnet’s different distance and angle with the ferrofluid. The sensitivity reaches 21.48 kPa^−1^ at region I and 1.14 kPa^−1^ at region II when D = 12 mm. The spike is the highest at this time, and the experimental results correspond to the theoretical analysis which is the higher the microstructure, the greater the sensitivity within a certain range. As for angle, the sensitivity reaches up to 15.21 kPa^−1^ at region I and 0.47 kPa^−1^ at region II at α = 30°. And the FTTS shows a fast response time (~ 90 ms), the minimum detect force is 1.25 Pa, also the oleophobicity of the solid–liquid interface can greatly reduce the wear and tear of FTTS, resulting in the improvement of stability. Last but not least, this sensor can be used as a new-type password lock. Through controlling the pressure level when inputting the password combination, the double protection of the password lock is formed. This brings absolute safe and reliable protection to the user and has great potential in smart home and IoT.

## Supplementary Information

Below is the link to the electronic supplementary material.

Supplementary file 1 (PDF 478 kb)

Supplementary file 2 (AVI 6633 kb)
