# Supplementary material for: A Liquid–Solid Interface-Based Triboelectric Tactile Sensor with Ultrahigh Sensitivity of 21.48 kPa−1
Source: Nanomicro Lett. 2022 Apr 1;14:88. doi: 10.1007/s40820-022-00831-7 (PMC8975924; doi:10.1007/s40820-022-00831-7)
Supplement: Supplementary file 1 — (PDF 478 kb) [file 40820_2022_831_MOESM1_ESM.pdf]

Supporting Information for

A Liquid-Solid Interface based Triboelectric Tactile Sensor with Ultrahigh Sensitivity of 21.48 kPa^{-1}

Jingya Liu[1], Zhen Wen[1,*], Hao Lei[1], Zhenqiu Gao[1], and Xuhui Sun[1,*]

[1]Institute of Functional Nano and Soft Materials (FUNSOM), Jiangsu Key Laboratory for Carbon-Based Functional Materials and Devices, Soochow University, Suzhou 215123, P. R. China

*Corresponding authors. E-mail: wenzhen2011@suda.edu.cn (Z. Wen); xhsun@suda.edu.cn (X. Sun)

Supplementary Figures

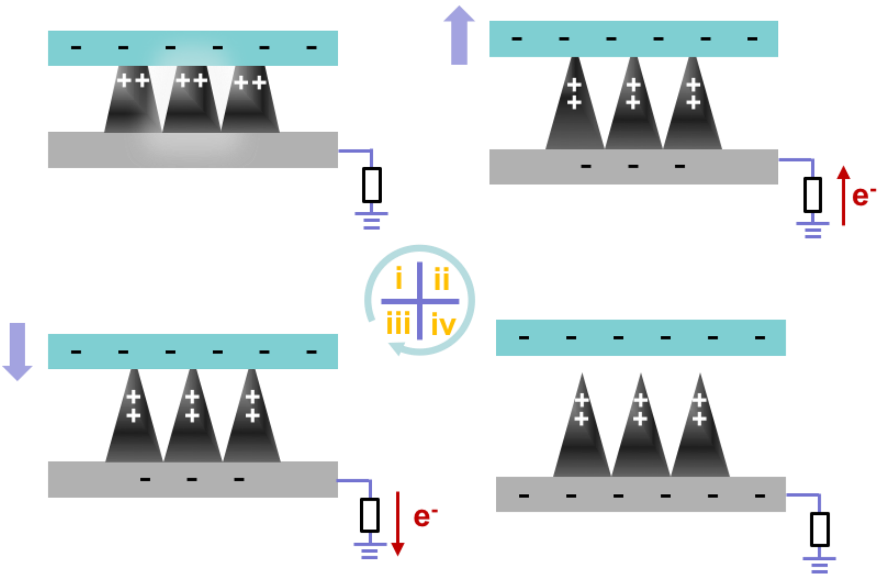

Fig. S1 Complete working principle of the FTTS

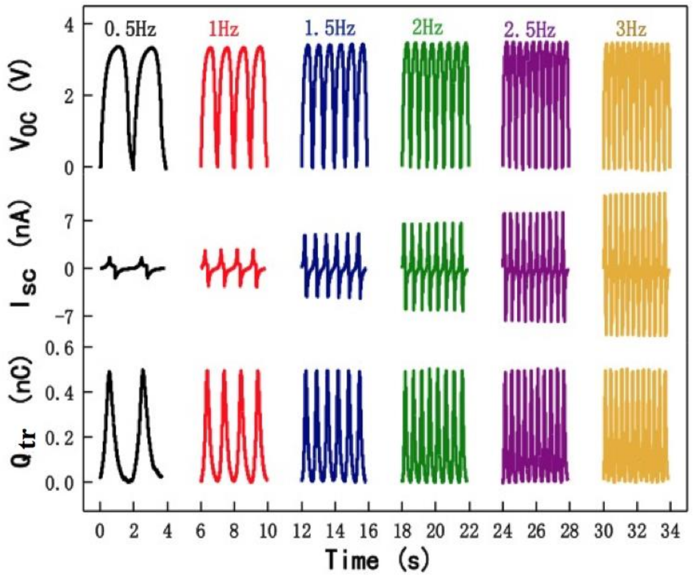

Fig. S2 Electrical output of the FTTS including open-circuit voltage (V_{oc}), short-circuit current (I_{sc}) and transfer charge (Q_{tr})

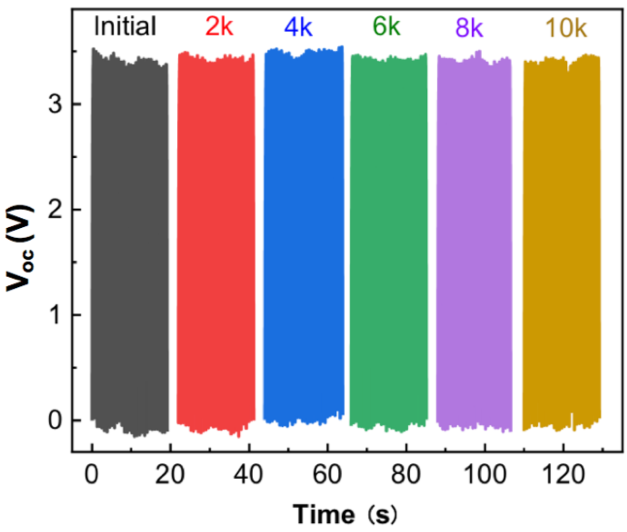

Fig. S3 Stability testing of the FTTS with 10000 cycles

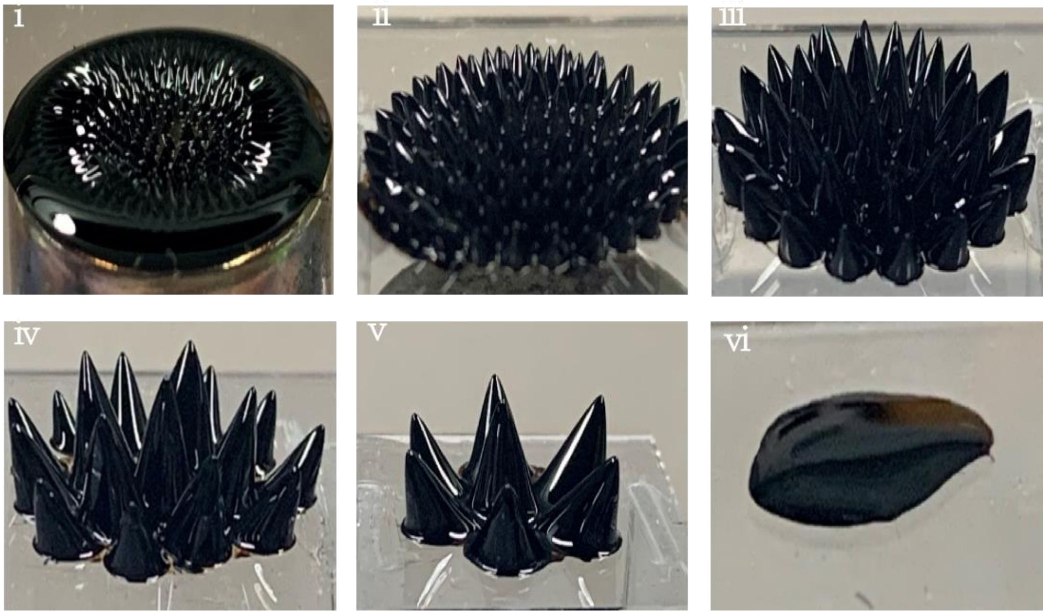

Fig. S4 Photograph of the topography of ferrofluid patterns at all six distances with magnet, from 0 to 2 cm at intervals of 4 mm

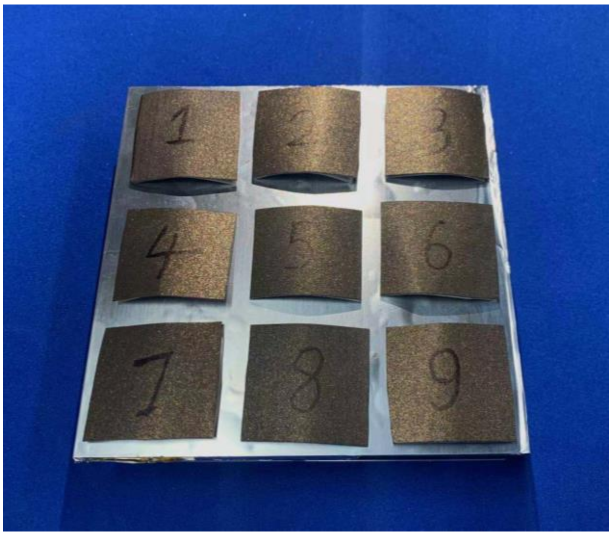

Fig. S5 Photograph of FTTS array as a nine-digit password lock

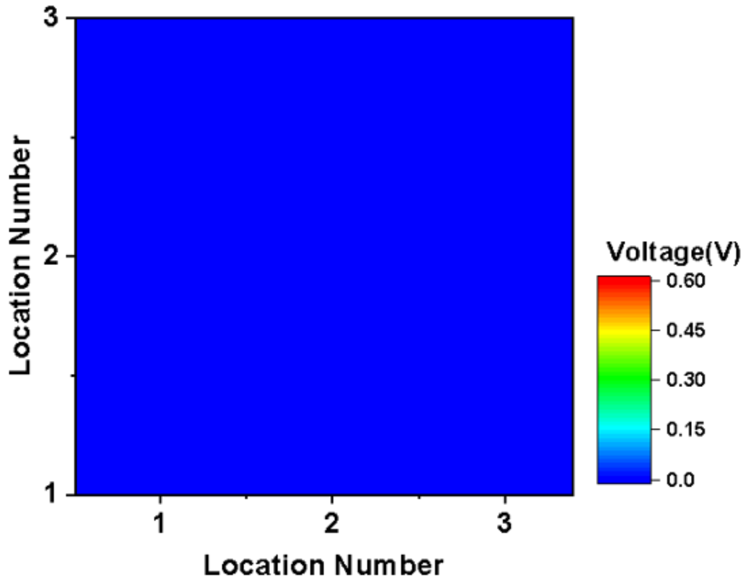

Fig. S6 Contour map of voltage output of all FTTS units in the matrix is at the background level before any pressing

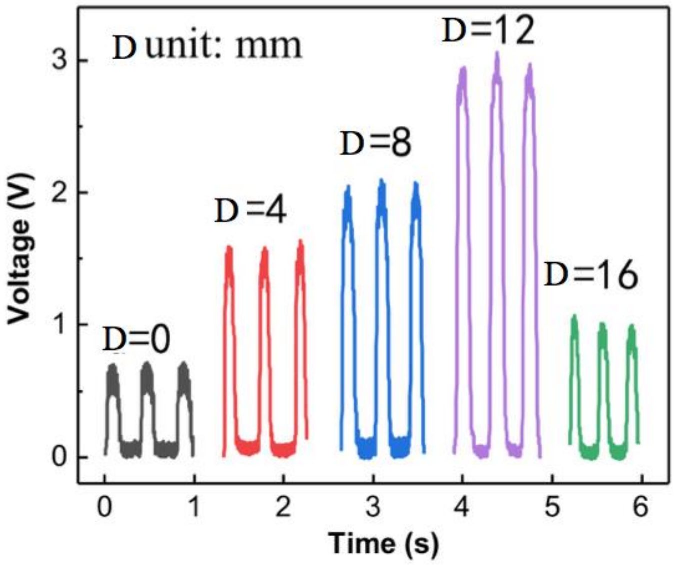

Fig. S7 Different voltage output when changing distance with magnet under the same external pressing (F = 20 N)
